# New Zealand women’s experiences of managing gestational diabetes through diet: a qualitative study

**DOI:** 10.1186/s12884-021-04297-0

**Published:** 2021-12-10

**Authors:** R. L. Lawrence, K. Ward, C. R. Wall, F. H. Bloomfield

**Affiliations:** 1 The Liggins Institute, The University of Auckland, Building 505, Level 2, 85 Park Road, Grafton, Auckland, 1023 New Zealand; 2 School of Nursing, University of Auckland, Auckland, New Zealand; 3 Faculty of Medical and Health Sciences, University of Auckland, Auckland, New Zealand

**Keywords:** Gestational diabetes, Dietary advice, Dietary behaviour, Patient experience, Qualitative research

## Abstract

**Background:**

For women with gestational diabetes mellitus (GDM) poor dietary choices can have deleterious consequences for both themselves and their baby. Diet is a well-recognised primary strategy for the management of GDM. Women who develop GDM may receive dietary recommendations from a range of sources that may be inconsistent and are often faced with needing to make several dietary adaptations in a short period of time to achieve glycaemic control. The aim of this study was to explore how women diagnosed with GDM perceive dietary recommendations and how this information influences their dietary decisions during pregnancy and beyond.

**Methods:**

Women diagnosed with GDM before 30 weeks’ gestation were purposively recruited from two GDM clinics in Auckland, New Zealand. Data were generated using semi-structured interviews and thematic analysed to identify themes describing women’s perceptions and experiences of dietary recommendations for the management of GDM.

**Results:**

Eighteen women from a diverse range of sociodemographic backgrounds participated in the study. Three interconnected themes described women’s perceptions of dietary recommendations and experiences in managing their GDM through diet: managing GDM is a balancing act; using the numbers as evidence, and the GDM timeframe. The primary objective of dietary advice was perceived to be to control blood glucose levels and this was central to each theme. Women faced a number of challenges in adhering to dietary recommendations. Their relationships with healthcare professionals played a significant role in their perception of advice and motivation to adhere to recommendations. Many women perceived the need to follow dietary recommendations to be temporary, with few planning to continue dietary adaptations long-term.

**Conclusions:**

The value of empathetic, individually tailored advice was highlighted in this study. A greater emphasis on establishing healthy dietary habits not just during pregnancy but for the long-term health of both mother and baby is needed.

**Supplementary Information:**

The online version contains supplementary material available at 10.1186/s12884-021-04297-0.

## Introduction

During pregnancy, a nutritious diet is important for both the mother’s health and the growth and development of the baby [1, 2]. Pregnancy is often referred to as a ‘teachable moment’ when women are amenable to dietary changes [3, 4]. However, some view pregnancy as a time when indulgence is acceptable, or even beneficial, as the pregnant woman is ‘eating for two’ [5]. Gestational diabetes mellitus (GDM) is a form of carbohydrate intolerance first diagnosed in pregnancy [6], which leads to hyperglycaemia and poses significant health risks to both mother and baby. Globally, the prevalence of GDM varies widely [7] with overall trends showing increasing prevalence [8]. In New Zealand, GDM is estimated to affect around 6% of pregnancies [9]. For women with GDM, poor dietary choices resulting in hyperglycaemia can have harmful consequences [10–13]. Diet is well recognised as the cornerstone of GDM management [14–16] and referral to a dietitian is recommended for all women diagnosed with GDM [17–22]. In New Zealand, women with GDM are referred to a multidisciplinary diabetes in pregnancy clinic for management of diabetes [21]. The structure of these clinics varies across New Zealand regions but typically include a diabetes physician, obstetrician, diabetes midwife or diabetes specialist nurse and a dietitian. Although studies have found dietetic input to be associated with positive outcomes for women with GDM [23–25] surveys of dietetic practice have identified some inconsistencies in the services provided and recommendations made to women with GDM with some women with GDM not seeing a dietitian at all [26–29]. Furthermore, women are exposed to nutrition messages from several sources which may be inconsistent or incomplete [30–33] leading to confusion about what to do and a lack of confidence in recommendations [34, 35]. GDM is usually diagnosed at 24 to 28 weeks’ gestation [6, 21]. Thus, women who develop GDM typically need to make several dietary adaptations in a short period to achieve glycaemic control [36–38]. This urgency to master self-management of GDM may be overwhelming for some [35, 36, 39, 40].

There have been international studies exploring women’s experiences of GDM, but few have specifically considered the experiences around dietary adaptations [35, 37, 40, 41]. As diet is central to a women’s management of GDM, a greater understanding of how women perceive dietary advice and how it influences their dietary decisions is important and could help healthcare professionals to tailor care to the needs of women diagnosed with GDM. In this context, we explored New Zealand women’s experiences of dietary recommendations following diagnosis with GDM from their perspective. We focused on how women diagnosed with GDM perceive the dietary information given to them and how this information influenced their dietary decisions during pregnancy and beyond.

## Methods

### Setting and sample recruitment

Participants were purposively recruited [42] from two large regional health boards in Auckland, New Zealand where the incidence of gestational diabetes at the time of recruitment was around 11% [43, 44]. A member of the GDM clinic team invited women to participate in the study if they were currently pregnant with a diagnosis of GDM made before 30 weeks’ gestation. Guidelines for the diagnosis and management of GDM in New Zealand at the time of the study recommend universal screening for diabetes in pregnancy at the first antenatal visit using HbA1c, with an HbA1c of 50 mmol/mol or above considered to indicate pre-existing undiagnosed diabetes, followed by further screening at 24–28 weeks’ gestation using either a 50 g glucose challenge test if their early HbA1c was ≤40 mmol/mol or a 75 g oral glucose tolerance test if early HbA1c was 41–49 mmol/mol [21]. A diagnosis of GDM is made if blood glucose values exceed ≥11.1 mmol/L after a 1 h, 50 g oral glucose challenge test, or if in a 75 g oral glucose tolerance test fasting glucose is ≥5.5 mmol/L or two hour blood glucose is ≥9.0 mmol/L [21]. However, some women present late for the GDM screening test and others are screened early where there is clinical concern. The cut-off for diagnosis before 30 weeks’ gestation was chosen after consulting with the lead physician and obstetrician at both district health boards. They both suggested a women’s care when diagnosed after 30 weeks’ gestation might be different to those diagnosed earlier in pregnancy, and 30 weeks would allow time to experience GDM prior to arranging an interview. Women with pre-existing diabetes mellitus, those under the age of 16 years and those unable to adequately understand verbal explanations in English or who had special communication needs were excluded. All women gave written or verbally recorded informed consent. Women were offered a $25 grocery voucher to thank them for their participation in the study.

### Data collection and analysis

Data were generated using semi-structured interviews asking about women’s experiences of managing GDM through their diet. A semi-structured interview guide (Table S1) was developed by a New Zealand Registered Dietitian with clinical experience in GDM (RL) and a qualitative researcher with a background in nursing (KW). Broad interview questions were used to allow women to describe their experiences in their own words. Prompts elicited further information where necessary. Interviews were conducted between August and December 2019 by RL, who was not involved in the women’s care. Participants chose to be interviewed over the telephone, in person at their own home, in a private meeting room or a private space at the GDM clinic. Before commencing the interview, women completed a short demographic questionnaire, which included questions relating to age, ethnicity, gestation, parity, history of GDM in a previous pregnancy and gestation at GDM diagnosis. The New Zealand Deprivation Index (NZDep2013) [45] was used as a measure of social deprivation using participants’ home address. NZDep 2013 groups deprivation scores into deciles where 1 represents the least deprived and 10 the most deprived 10% of areas in New Zealand.

Interviews were audio-recorded and transcribed verbatim by RL. Women either chose their own or had a pseudonym allocated to them to preserve their anonymity in the transcripts and reporting of data. Reflexive thematic analysis was chosen as a pragmatic and flexible approach to analysis that is data-driven and not tied to a pre-existing coding framework [46, 47]. RL independently coded the data through repeated readings of the transcripts using a general inductive, experiential approach. Ideas or issues raised by participants supported code development. Codes were then grouped into themes that comprised codes of shared meaning connected through a central concept for example, Managing GDM is a balancing act [47, 48]. Codes and themes were then discussed with KW, an experienced qualitative researcher, to refine and confirm themes as an authentic reflection of the participants’ words.

Data collection and analysis occurred concurrently to allow adaptation of interview questions to follow leads in the data based on information from each subsequent participant. Interviews continued until data saturation was achieved. Data saturation was the point at which, after reviewing coding and theme development from previous interviews, RL and KW agreed that further interviews were unlikely to reveal new information [49]. Two further interviews were conducted to confirm that data saturation had been achieved.

This study was approved by the Auckland Health research Ethics Committee (reference 000121) and reported according to the consolidated criteria framework for reporting qualitative studies (COREQ) [50].

## Results

A total of 18 women participated in the study. Women were a median age of 34 years (range 28 to 41), from a range of ethnic backgrounds, and half (*n* = 9) were expecting their first baby (see Table 1). Most women (*n* = 15) were experiencing GDM for the first time and were diagnosed with GDM for a median of 9.6 weeks (range 4.0 to 21.7 weeks) before the interview. Half (*n* = 9) of the interviews were conducted in person and half (n = 9) over the telephone. Interviews lasted a median of 45 min (range 21 to 96 min).

**Table 1:** Participant and interview characteristics from the Managing gestational diabetes through diet study (*n* = 18)

Participant (pseudonym)	Age	Ethnicity	Socioeconomic deprivation (NZDep2013)	Parity	GDM in previous pregnancy	Gestation at diagnosis (weeks)	Gestation at interview (weeks)	Interview mode	Interview length (minutes)
Amipa	36	Tongan	1	3	Yes	28	35	Telephone	40
Deepti	33	Indian	4	0	–	28	32	Telephone	54
Evergreen	35	Korean	9	0	–	16	37	In-person	32
Fei	36	Chinese	9	1	No	12	21	Telephone	22
Gretchen	32	German	4	0	–	24	31	In-person	72
Huian	35	Chinese	5	1	No	28	37	Telephone	31
Jing	35	Chinese	1	1	Yes	11	24	Telephone	29
Juliana	41	Brazilian	5	0	–	28	36	In-person	37
Kate	30	NZ European	2	2	No	29	35	In-person	68
Mai	35	Vietnamese	10	1	No	26	33	In-person	59
Marama	40	Māori	6	1	No	22	35	Telephone	43
Moeroa	29	Cook Islands Māori	10	0	–	27	31	In-person	34
Nian Zhen	35	Chinese	8	0	–	11	32	In-person	96
Rachel	33	NZ European	8	0	–	26	31	Telephone	33
Rosa	31	Filipino	7	1	No	26	37	Telephone	38
Rose	31	Chinese	2	1	Yes	25	34	In-person	63
Seini	28	Tongan	10	0	–	25	36	Telephone	33
Vishakha	33	Indian	7	0	–	12	28	In-person	29

**Fig. 1:**
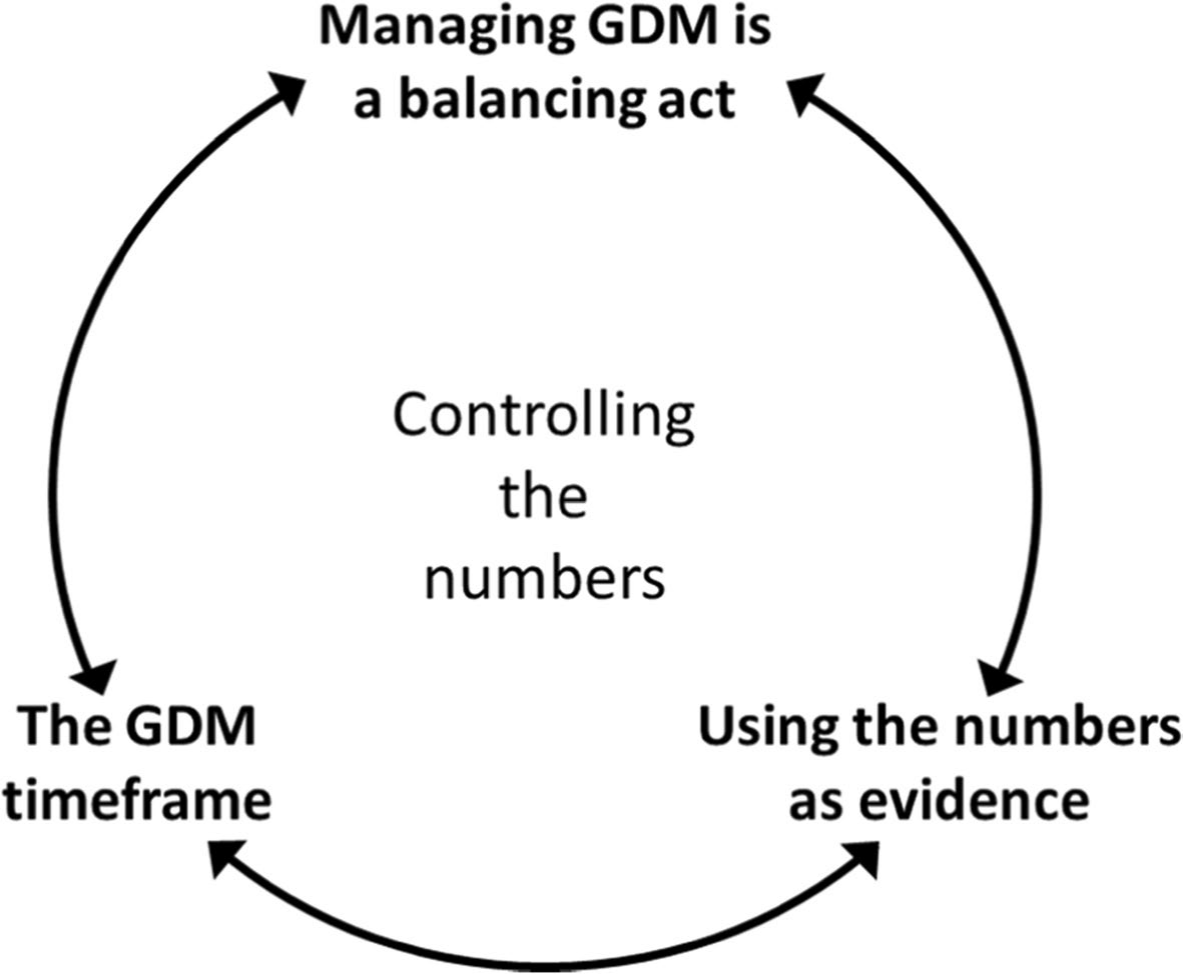
Themes describing women’s experiences of managing GDM through diet

Overall, women described the primary objective of dietary advice to be to control their blood glucose levels and controlling the numbers was the focus of many women’s story. Thematic analysis yielded three intricately intertwined themes that encompassed women’s experiences of dietary recommendations and managing their diet to achieve the objective of controlling the numbers (Fig. 1).

Women described *managing GDM as a balancing act* wherein they needed to balance their diet and all the demands of their existing lives with the need to keep their blood glucose readings within the recommended range. Women described using their blood glucose results or *numbers as* “*evidence*” (Nian Zhen): evidence that they really did have GDM or that how they were eating was acceptable or not. Participants frequently described GDM and the dietary adaptations they made to manage this in the context of time or within *the GDM timeframe.* Each theme is described in detail below using participants excerpts to evidence the themes.

### Theme 1: managing GDM is a balancing act

After receiving their diagnosis of GDM, women described needing to find a balance or a way of fitting in the diagnosis and management of GDM with their existing life and preconceived conceptions and understanding of a healthy diet. For some, there was also a need to reconcile their expectations of pregnancy with now having a pregnancy affected with GDM.

Many women described being “shocked” by their diagnosis of GDM despite having risk factors for its development (Amipa, Evergreen, Fei, Mai, Rachel). Even if they were aware of these risk factors, some simply did not believe it would “happen to them” (Fei, Marama, Nian Zhen). Evergreen described the isolation she felt after receiving her diagnosis. She felt she had no one to talk to about having GDM as her Korean cultural upbringing made the subject taboo:

“my culture background, they don’t really tell much about your personal health… For me I feel very alone, because I no one talk, you know. There’s only the health professional, that’s only person I can rely on.”

Some women spoke about grappling with the change in their pregnancy identity as there was a sense that having GDM meant their pregnancy was now “different” (Deepti). There was an undertone of injustice for some women as they felt pregnancy was supposed to be a time where they could indulge and give in to their pregnancy cravings. Gretchen, who was in her first pregnancy, described her feelings of having a GDM affected pregnancy as follows:

“first of all it’s the first pregnancy, and second everyone’s saying yeah you have to enjoy that, that’s an awesome time and the first thing that you do is to check your blood sugars, you have to check what you’re eating, and that’s not fun, so it’s far away from enjoying the feeling of being pregnant.”

Women also expressed feeling alone in their diagnosis as their family or other pregnant women did not have to make the same sacrifices (Jing, Seini, Nian Zhen). Seini spoke of both views saying, family “can be supportive and then at the same time you’re kind of on your own… they’re eating something different”.

Several women described difficulty fitting in the way in which they now needed to eat to manage their GDM with their family norms or expectations:

“mum is typical Indian and she just like sometimes we can eat other stuff but mum eats roti. So she needs Indian food… it is like hard work because I usually have to cook same food for everyone. Now I have to cook mine different, and my family’s different.” (Deepti)

Nian Zhen, who had been through some years of fertility treatment, reported a real shift in her priorities at home to manage her GDM, saying she did not “care much about [her] husband taste of food anymore, I just make sure that I have the food, which is gonna be healthy for my baby.” Others found a way of balancing what they needed to do to manage GDM whilst still fitting in with the rest of the family. Marama described eating the same as her family, “except when I buy my sugar-free stuff, they’re not allowed it.” Kate described her accommodation as:

“I haven’t altered really what I make. Like we all eat the same thing, but if I’ve made like, curry and rice for instance, I’ll go heavier on the curry and less on the rice, than everyone else will. I don’t want to be making stuff that’s not healthy for everyone else just because they don’t have gestational diabetes.”

A number of women described a degree of dissonance between the information they received from healthcare professionals and their pre-existing beliefs or what they had researched themselves. Some described needing to find a middle-ground or “strike a happy balance” (Rosa) when they were not entirely comfortable with the information received. Others, over time, came to accept that the information provided was correct or in their best interests. Marama said, “I didn’t think I was that bad of an eater. And I thought, I, I didn’t [need to] make as much changes as I did. But yeah. Not that healthy after all!”

Some women struggled with the advice they had received, particularly with fitting it in with their existing lifestyles or cultural norms and reported feeling that the advice was not particularly “relevant” (Kate) to them as an individual or to their culture. Amipa felt the information she received was “not for a Pacific woman.” Rose added:

“I think some of the food like I can’t, like I never tried… That, like they recommend oh this is you know, combined with vegetables and blah blah and uh this is balanced diet, I say but you know we eat rice.”

Indeed, Amipa, in her second pregnancy affected by GDM, reported seeking and receiving more relevant and practical information from her Pacific Island community. Similarly, Rosa, a Filipino women in her second pregnancy, did not fully agree with the advice she received from healthcare professionals, finding information that better suited her from a book, which made more “sense” to her and gave “options” that were closer to her pre-existing diet.

A women’s relationship with the healthcare professionals caring for them appeared to influence how they perceived the dietary advice provided and how supported they felt in managing their condition. Some women felt “special” (Vishakha) and genuinely cared for by their healthcare professional. This was particularly evident when women were in close contact with their diabetes midwife or followed up regularly by other healthcare professionals. Juliana described feeling “safer now” because “now that I have this direct contact with the diabetes midwife, it’s easier because everything that I eat or if I have some question or anything I just email her and she emails me back.”

On the other hand, some women felt like they were “just a number” or “another pregnant woman” to get through (Amipa). It seemed women reported feeling this way when healthcare professionals were not empathetic towards their personal circumstances, details about their care were forgotten, or advice was not tailored specifically to them. Amipa described two very different experiences with two different healthcare professionals:

“So you’ve just been told you need to do this and this and that and that and then show up to this and that alright then bye. There’s nothing really special about you as an individual… I was telling [the diabetes midwife] about what is my diet and she was just like oh yup yup, yup, umm, okay so this is what we’re gonna do, this is umm, yeah she wasn’t really listening like the dietitian that I saw. The dietitian was really good, she was actually really listening, she gave me some feedback, she gave me ideas like how I could change things around which I did.”

One factor that seemed to help women find balance in managing GDM was being involved in making decisions about their care and management. Some women even reported feeling trusted in this way and this strengthened the relationship between the woman and healthcare professional. Vishakha described having the “freedom to do so because they could see that I’m actually being honest about my sugar levels.”

### Theme 2: using the numbers as evidence

In many ways, women used their blood glucose results as evidence. Evidence that they did have GDM after their initial disbelief that it would happen to them. Evidence that they were doing a good job or “behaving” (Nian Zhen) in terms of their diet. Evidence that the advice provided by healthcare professionals could be trusted, and evidence as to whether they would need to continue to control their diet in the future.

Marama reported thinking her diet “wasn’t that bad at first” but after seeing consistently high results in her blood glucose monitoring, she felt this was evidence that she had to start taking her diagnosis “seriously”. Once women accepted the need to control their blood glucose, this became the focus of their pregnancy. Women felt the constant need to monitor their blood glucose results made them more “conscious” (Seini) and accountable for what they chose to eat. Universally, women described their motivation to manage GDM as being to ensure the wellbeing of their baby, but the need to control their blood glucose readings became so dominant that the result on their blood glucose meter was frequently their first thought or primary focus, rather than the potential consequences for their baby. Amipa and Rachel both described refraining from indulging because of the possibility of a high reading at their next blood glucose check:

“‘Cos you know like in two hours’ time you’re going to be checking your sugar levels, so you can’t really go pig out on the chocolate cake.” (Amipa)

“I don’t want to get a seven or an eight on my blood test after dinner so I’m not going to eat that.” (Rachel)

Some women described using their blood glucose readings as evidence that it was acceptable to “cheat” or “sneak in” (Rosa, Gretchen) foods they felt were technically off limits. If the results were still within the recommended range, then this was deemed acceptable:

“after checking the sugar, that’s when I sneak in my, my cheat snack. And it comes with my dinner and then it gets check after dinner with my sugar so far, with everything that I’m eating it’s all been maintained.” (Rosa)

Similarly, Deepti described using her blood glucose readings to experiment with food as a way to increase the variety of her diet, “the very first week was up and down because I trying what I can eat.”

On the other hand, women reported feeling frustrated when their blood glucose readings were high for no apparent reason despite their efforts. Kate relayed, “I’ll eat the same thing and sometimes the sugar’s great and sometimes it’s high and I’m like *why* it makes no sense!”

Women also used numbers as evidence that they could trust the healthcare professionals caring for them or the advice they were receiving was trustworthy. Marama reported, “I trust them now” after seeing that the times where she followed healthcare professionals’ recommendations were “matching up with when my blood sugars are good.” Conversely, Nian Zhen reported being given a pamphlet by the dietitian that she “can’t follow” because of high blood glucose readings after consuming the foods listed. She went on to say the glucometer was the only thing she could trust because it would not “cheat on” her.

### Theme 3: the GDM timeframe

Timing and the timebound nature of pregnancy and GDM featured in many women’s narrative. For many women managing GDM involved sticking to a timetable of eating meals and testing blood glucose levels at certain times of the day and that this was something you “got used to” over time (Gretchen, Amipa, Deepti, Moeroa). For some, the changes they made to their diet to manage GDM was made easier by knowing there was “an end to it” (Juliana, Kate, Nian Zhen, Rose) once their baby was born, while others hoped to continue with the changes to their diet in the future.

On receiving her diagnosis, Juliana, a 41-year-old woman who described difficulty in conceiving, recalled feeling as though she “had to do something quick” to get her blood glucose levels under control immediately, “from that day I decided not to have more umm, treats and cakes and pastries”. She felt the timing of her GTT and the period in which she had to wait for a clinic appointment or advice “took too long”. She reported feeling “scared” during the weeks she waited for a GDM clinic appointment and worried about how it was affecting her pregnancy.

Women frequently described sticking to a timetable or “routine” (Rosa) in terms of when they ate and when they tested their blood glucose levels as a way in which they managed having GDM. However, some found this need for regularity difficult to fit in with their existing lifestyles. Huain described finding it “difficult” to have her meals “at the same times every day.” Similarly, Mai reported being told to “have three main meals a day and snacks between the meals” but found this challenging to fit into her daily routine and “to keep enough time gap between the meal and snacks so that it doesn’t impact the readings.”

Although initially managing GDM through diet was described as a steep learning curve for many women, on the whole, women reported they “get used to it” (Amipa, Deepti, Gretchen, Marama, Moeroa) and gain confidence and competence over time. Many reported that only needing to follow recommendations for the duration of their pregnancy made it easier to comply with the restrictions placed on their diet. Juliana said “at least I hope, there’s a deadline. I know that it’s going to end. So, it’s easier to manage.” Similarly for Kate, watching those around her consume foods and drinks she was trying to avoid was made easier because “I’ve just got a few more weeks of this” conversely she stated, “if it was the rest of my life, I’d probably be a bit more like, how ‘bout you drink that while I’m not watching.”

The understanding that their actions in the short-term, during pregnancy, could have long-lasting effects on their baby was also a strong motivator for women. Nian Zhen commented, “if you don’t behave now, it will be bad impact for the [baby]. So, I don’t want that happen. I just suffer 10 months, that will be like the forever years for the [baby].” Similarly, Rachel stated, “if what I’m eating now can affect [the baby], I can cut out sugar, I can cut out white bread, because you know, it could be a lifelong issue for my child if I don’t.”

A few women described feeling as though they were simply a vehicle for the baby’s health and that the healthcare practitioner’s primary concern was not for themselves but for the baby. Rose relayed a conversation she had with her midwife demonstrating this:

“The midwife says you know, if you want to eat this you can eat it after you have the baby, but now, what we’re doing here is for your baby.”

When healthcare professionals’ focus was on the baby, women reported feeling as though they were “off the hook” or on their own after pregnancy (Rose). Many women talked about relaxing their diet after pregnancy despite their increased risk of developing type 2 diabetes, as their dietary choices would only affect their own health and not that of their baby. However, for some this would be dependent on the *numbers as evidence* that it would be acceptable to do so. Huian stated, “I’ll start eating as usual if umm my sugar level is back to normal” while Nian Zhen planned to continue her dietary restrictions until she “passed” the postnatal blood test, after which she planned to “be crazy naughty again.”

Other’s took their diagnosis and the advice provided to them as a “wake-up call” (Seini) or “opportunity to reflect” (Fei) on their current lifestyle and make changes to reduce their risk of type 2 diabetes in the future. Moeroa saw her diagnosis of GDM as a learning opportunity not just for her but for her wider family as well:

“My family they’re big eaters as well, just showing them and role modelling like hey there’s certain foods we can eat. It’s good so that can support them later in the future you know and if they find out they got it [diabetes] then I can just help them out.”

## Discussion

Findings from this exploratory study have provided an understanding of New Zealand women’s experiences of dietary recommendations following a diagnosis of GDM. Overall, participants in this study perceived the primary purpose of dietary advice to be to control their blood glucose levels. Based on the experience of dietary advice, participants managed GDM by balancing the numbers as evidence within their perceived timeframe of consequences from GDM to themselves and their baby.

Whilst some women acknowledged that the prescribed dietary changes equated to a healthier diet, few spoke of receiving dietary advice to ensure a healthy pregnancy or optimise their health; rather, the focus was on maintaining blood glucose levels within limits. One other New Zealand qualitative study exploring barriers and enablers to achieving optimal glucose control in GDM also reported blood glucose results or “numbers” as the focus of women having GDM [51]. Women in that study reported feeling as though their blood glucose results “ran their life” and did not enjoy the shift in focus of their pregnancy to their blood glucose “numbers” [51]. Women in our study reported feeling as though their pregnancy was now “different” because of GDM, consistent with other reports [52–54]. Indeed, midwives caring for women with GDM have expressed that, once a woman was diagnosed with GDM, her pregnancy was no longer considered normal [55]. These findings may validate women’s feelings of having a ‘different’ or ‘medicalised pregnancy’ [53, 56]; and is supported by women reporting that once they were diagnosed with GDM, their diabetes became the focus of medical attention rather than their pregnancy [56]. In our study, some women described feeling that healthcare professionals had greater concern for the baby than for the women themselves, with the focus on numbers making them feel as though they were merely a vehicle for the baby’s health. These findings are consistent with a study in 2018 in which women described feeling that the hospital “claimed ownership of the baby”, that healthcare professionals were now in control of their pregnancy, and that they felt viewed objectively rather than personally and as a “possible obstacle to the baby’s wellbeing” [38].

Women with a history of GDM who were in their postpartum period reported feeling abandoned once they had delivered their baby despite their increased risk of type 2 diabetes [38, 57–59]. This postpartum abandonment may compound women’s feelings of being unimportant and simply “baby machines” and may lead to feelings of ambivalence to their own health [38]. A low perception of future risk of type 2 diabetes in a study of 35 women with a history of GDM in South Africa was attributed to the focus on the health of the baby and blood glucose results during pregnancy and subsequent “abandonment” postpartum [58]. We encountered similar feelings with women in our study reporting feeling that after pregnancy they were “off the hook” and could relax dietary restrictions or even “go crazy”, as what they ate would no longer affect their baby’s health, even though they were aware they would be at risk of type 2 diabetes. We argue that the fetal-centric approach many women described experiencing during pregnancy may negatively impact women’s motivation to continue healthy dietary habits that may reduce the risk of type 2 diabetes in the future. Similar findings have been reported in the literature on interventions aimed at smoking cessation during pregnancy [60], in which many women who quit smoking during pregnancy resume smoking within the first year postpartum. Our data indicate that dietary advice received seemed to have the greatest influence on participants’ dietary decisions in the short-term. Only a few women reported viewing the information as beneficial for their long-term health or wanting to role model good eating habits for their families. Other studies report that although following a healthy diet and lifestyle was a concern for the whole family during pregnancy, other priorities took over once the baby was born [59, 61]. However, mothers’ dietary habits, attitudes and beliefs about food and nutrition are thought to have a significant influence on their children’s dietary behaviours [62–64]. These findings indicate that healthcare professionals should consider and highlight the importance of healthy behaviours for both mother and baby, and continuing healthy behaviours established during pregnancy both for the future health of the women themselves and and of their infants.

Lack of culturally-tailored dietary advice reported in our study and others may pose a barrier for achieving and continuing with dietary behaviour change [38–40, 65]. Women from ethnic minorities often experience higher prevalence of GDM [66, 67] and may face additional challenges in understanding and managing the condition [39, 68–71]. A systematic review of studies involving women with a history of GDM proposed that dietary advice not fitting with a woman’s cultural identity may make changes more difficult to sustain long-term [72]. Healthcare professionals need to acknowledge that food is more than simply energy and nutrients. Food plays an important role socially and culturally and, for many women, GDM significantly reduces the enjoyment, spontaneity, social and cultural aspects of food [71]. A woman in the study by Svensson et al. (2018) felt the imposed dietary restrictions took away the “joy of pregnancy” [59], a sentiment echoed by several women in our study and in others [37, 73]. Women described experimenting or “cheating” with different foods as a way of teaching themselves which foods could fit into their GDM ‘diet’ or as a way to retain some pleasure in their diet. However, for many it was clear that in the initial phases of experimenting, there were a number of blood glucose levels above recommended limits. Women described these as acceptable as they were used to increase the variety of their diet or satisfy their cravings, but few recognised the impact these excursions may be having on their baby. Similar behaviours are commonly described in the literature [39, 53, 56, 74]. Rather than an act of non-compliance, women described this as a way of minimising the intrusiveness of GDM on their lives [56]. Perhaps if women felt better supported with the dietary adaptations through more individually tailored advice, the need to experiment or “cheat” would be reduced.

A key mediator of women’s perceptions of dietary advice in our study was their relationship with the healthcare professional. Being treated with empathy and regular contact or follow-up with healthcare professionals had a positive effect on a woman’s relationship with healthcare professionals. When women felt healthcare providers lacked empathy or did not consider their individual circumstances, women implicitly and explicitly reported feelings of mistrust towards healthcare professionals and sought information from alternative sources. Other studies report similar findings [37, 38, 73] and that feelings of connection between women and healthcare providers positively influence women’s perception of the quality of care and their adherence to treatment [61]. Midwives in Sweden have reported employing a range of different strategies to manage their encounters with women. When unsuccessful in establishing an empowering relationship, wherein they work in partnership with women to manage their GDM, some midwives resort to a paternalistic approach as they feel they have a duty to protect the baby’s health [55]. However, this paternalistic approach has potential to lead to paradoxical results. Reactance theory proposes that interventions that pose a perceived threat to a patient’s freedom can lead to non-compliance with recommendations [75]. This may explain the behaviours of some of the women in our study and others’ such as ‘cheating’ or seeking alternative sources of information [37, 38]. Women’s relationships with healthcare professionals during pregnancy has also been reported to influence their engagement with postpartum follow-up and health behaviours after pregnancy [76, 77]. The way in which healthcare professionals interact with women with GDM, rather than just the provision of dietary recommendations, can therefore play a significant role in the women’s management of GDM and long-term health outcomes. Greater recognition of the psychological and long-term health needs of women with complications during pregnancy have led to calls for a more comprehensive, woman-centred life-course approach to maternal health [78, 79].

### Strengths and limitations

A major strength of this study is the diversity of participants. Participants came from a range of ethnic and social backgrounds and represented a wide range of views and experiences. Open-ended questions were used to capture women’s experiences in their own words. Whilst interview questions focused on dietary recommendations, participants were free to share anything relating to their experience at the end of the interview. Recall bias was minimised by conducting the interviews whilst the women were still pregnant. Respondent burden and self-selection bias were reduced by offering women the choice to be interviewed in person, in their own home or at a location close to their GDM clinic site, or over telephone. Whilst some may suggest there is potential for differences in results obtained over the telephone compared to in-person [80] others have found this not to be the case [81, 82] and allowing participants to choose their preferred mode of interview may have minimised any effect. As with studies similar to ours, the generalisability of findings may be limited. Participants were recruited from two sites in Auckland, New Zealand, included only women who could converse in English and who were diagnosed before 30 weeks’ gestation. Our findings may therefore not be representative of all women diagnosed with GDM in New Zealand or be applicable to other countries where the model of care and experiences of women may be different. However, a systematic review of the psychosocial experiences of women with a diagnosis of GDM found common experiences among a diverse range of women from different countries [83] and our findings are consistent with those of studies including women from other populations. Our findings are valuable in providing insight into women’s experiences of dietary recommendations for GDM and may offer opportunities to adapt the way in which healthcare professionals interact with women with GDM in order to improve their experience and outcomes.

## Conclusions

On receiving a diagnosis of GDM, women are faced with a new challenge in navigating through their pregnancy to ensure the delivery of a healthy baby. The dietary advice women received to manage their GDM was primarily perceived as a means to control their blood glucose results for the sake of the baby. The women’s relationships with healthcare providers had a significant impact on whether women viewed recommendations positively or negatively. The value of an empathetic healthcare professional who recognises the significant impact GDM can have on a woman’s lifestyle, wellbeing and sense of autonomy was demonstrated in the narratives of women in this study. Individually-tailored, culturally appropriate advice and a greater emphasis on the woman with GDM, rather than just “the numbers”, is needed. Healthcare professionals should facilitate the establishment of healthy dietary habits not just for the duration of pregnancy but for the long-term health of both mother and baby. Further research on the experience and attitudes of healthcare professionals caring for women with a diagnosis of GDM could be useful in informing strategies to optimise the healthcare provider-patient relationship and provision of care for women with GDM.

## Supplementary Information

**Additional file 1: Table S1**. Semi-structured interview guide; semi-structured interview guide and prompts developed for the study.

## Data Availability

The data that support the findings of this study are held by the University of Auckland and have not been made available in order to preserve the anonymity of participants and healthcare professionals involved in their care. The semi-structured interview guide is included as a supplementary file. The study protocol can be made available upon reasonable request from the corresponding author.

## Abbreviations

COREQ: Consolidated criteria framework for reporting qualitative studies
GDM: Gestational diabetes mellitus
KW: Kim Ward (author)
NZDep2013: The New Zealand Deprivation Index
RL: Robyn Lawrence (author)
