# Supplementary material for: New Zealand women’s experiences of managing gestational diabetes through diet: a qualitative study
Source: BMC Pregnancy Childbirth. 2021 Dec 10;21:819. doi: 10.1186/s12884-021-04297-0 (PMC8662890; doi:10.1186/s12884-021-04297-0)
Supplement: Supplementary file 1 — Additional file 1: Table S1. Semi-structured interview guide; semi-structured interview guide and prompts developed for the study. [file 12884_2021_4297_MOESM1_ESM.docx]

**New Zealand women’s experiences of managing gestational diabetes through diet: A qualitative study**

**RL Lawrence, K Ward, CR Wall, FH Bloomfield**

| Table S1. Semi-structured interview guide | |
| --- | --- |
| **Key question** | **Related prompt question** |
| Can you tell me a little about your pregnancy so far? |  |
| How did becoming pregnant affect what you eat? |  |
| I understand that you’ve been told you have pregnancy related diabetes – tell me about that. | - What changed when you found out you had diabetes? - What thoughts did you have when you found out you had diabetes? |
| What has having diabetes meant for how you eat now? | - Tell me what kind of things you’ve changed since knowing about your diabetes. - What has gone well around choices you’ve made about food? - Tell me about a time when things have not gone well. - Tell me about a typical mealtime for you / and your whānau. - What thoughts go through your mind when you are preparing food for yourself / your whānau - Tell me how you imagine things will be once baby has arrived. |
| How have you found out about foods to eat for diabetes? | - What advice were you given? - How do you feel about this advice / what do you think about this advice? - What differences did it make speaking with a dietitian / other health professionals/ reading information online / listening to whānau / friends - What information was most helpful? - What information was least helpful? - What would you change about the way in which you were given this information? |
| Is there anything else you would like me to know? |  |
